# The Analysis of Classification and Spatiotemporal Distribution Characteristics of Ride-Hailing Driver’s Driving Style: A Case Study in China

**DOI:** 10.3390/ijerph19159734

**Published:** 2022-08-07

**Authors:** Runkun Liu, Haiyang Yu, Yilong Ren, Shuai Liu

**Affiliations:** 1 School of Transportation Science and Engineering, Beijing Key Laboratory for Cooperative Vehicle Infrastructure Systems and Safety Control, Beihang University, Beijing 100191, China; 2 Beijing Advanced Innovation Center for Big Data and Brain Computing, Beihang University, Beijing 100191, China

**Keywords:** driving style classification, ride-hailing drivers, trajectory data, temporal and spatial analysis

## Abstract

Monitoring the driving styles of ride-hailing drivers is helpful for providing targeted training for drivers and improving the safety of the service. However, previous studies have lacked analyses of the temporal variation as well as spatial variation characteristics of driving styles. Understanding the variations can also help authorities formulate driver management policies. In this study, trajectory data are used to analyze driving styles in various temporal and spatial scenarios involving 34,167 drivers. The k-means method is used to cluster sample drivers. In terms of driving style time-varying, we found that only 31.79% of drivers could maintain a stable driving style throughout the day. Spatially, we divided the research area into two parts, namely, road segments and intersections, to analyze the spatial driving characteristics of drivers with different styles. The speed distribution, the acceleration and deceleration distributions are analyzed, results indicated that aggressive drivers display more aggressive driving styles in road segments, and conservative drivers exhibit more conservative driving styles at intersections. The findings of this study provide an understanding of temporal and spatial driving behavior factors for ride-hailing drivers and offer valuable contributions to ride-hailing driver training and road safety management.

## 1. Introduction

Ride-hailing services have become popular in China [1]. In the service model, passengers use mobile apps to render ride-hailing services. Ride-hailing platforms, such as Didi Chuxing and Uber, distribute orders. Studies have shown that ride-hailing services can reduce detours and passenger waiting times and have the potential to improve traffic conditions and increase vehicle utilization [2,3,4]. As China’s largest online car-hailing service platform, Didi Chuxing has more than 31 million registered drivers and daily orders exceeding 60 million.

However, most ride-hailing drivers on the platform are not professional taxi drivers and are generally part-time [5]. These drivers do not go through unified, professional prejob training and strict operational qualification review; the driving styles of drivers are quite different, and the supervision is inadequate. Monitoring the driving styles of ride-hailing drivers and providing driving advice to drivers with a higher driving risk could be helpful for improving the safety of ride-hailing services and reducing fuel consumption [6,7,8,9,10,11]. Some studies have analyzed the factors that contribute to the driving risk of ride-hailing drivers. For example, ref. [12] believed that reckless driving will have an impact on driver safety risk. Ref. [5] found that the number of years of being a ride-hailing driver and the driving distance are significantly associated with the crash risk. These factors are due to different driving styles and driving skills [13,14]. This means that different driving styles can lead to different risk levels. Classifying the driving styles of drivers is an important way to identify high-risk drivers and help ride-hailing platforms provide targeted driving training for drivers with higher risks.

Research on the classification of driving styles has been extensive [15,16,17,18,19,20,21,22,23]. Previous studies have used different kinds of data, mainly experimental data, questionnaire data, and detector data. Additionally, multiple methods have been proposed to classify driving styles, such as clustering models, matching learning methods, and parameter threshold methods. Generally, drivers are divided into aggressive, general, and conservative types according to different driving styles [15,24], and the driving parameters of drivers with different driving styles are analyzed [17].

However, we found that in most studies, time-varying characteristics were not considered when analyzing driving styles. This is not suitable for ride-hailing drivers. Ride-hailing platforms need to evaluate the driving styles of drivers in real time and provide more or fewer orders based on the driver’s safety factor (https://www.wired.com/2016/01/uber-using-phone-data-to-track-how-fast-drivers-are-going/, accessed on 26 January 2016). For example, driver A passes through the intersection at a speed of 80 km/h during the peak period, and driver B passes through the road section at a speed of 80 km/h during the peak period. Obviously, these drivers have different levels of risk, and driver A is riskier than driver B. The ride-hailing platform should monitor driver A in detail and even reduce the number of his or her allocated orders. A lack of tracking time-varying driving styles and understanding spatial characteristics will prevent platforms from developing personalized driving management policies.

To fill these gaps, connected vehicle trajectory data are used to classify driving styles of ride-hailing drivers. The variability of driving styles is captured with changes in time, and the changes in driving parameters of different styles of drivers in space are analyzed.

### 1.1. Data Used for Driving Style Classification

There are three main sources of data for this study field. First, experimental methods are adopted to collect data [23,25,26,27,28,29,30]. For example, driving simulators are used to collect driving behavior data. The advantage of this method is that it can comprehensively monitor the driver’s driving state. In addition, corresponding questionnaires can be designed to assist the analysis. The sample size is generally small, and it is also convenient to label the driver category. However, the simulated driving environment cannot completely restore the real driving environment. Ref. [20] experimented with test drivers driving on roads, but the sample size was only 8, and the results may be biased. Second, a questionnaire survey was used to obtain drivers’ assessments of their driving styles [31,32]. However, this method is subjective, and the authenticity of the driver’s response to the questionnaire is biased. Third, the data were recorded by various types of detectors, such as global positioning systems (GPSs) and mobile phones, in a real driving environment [10,15,17,24,33]. Data collection is low in cost and large in quantity, and most importantly, these data can reflect the driver’s actual driving state. In this study, the trajectory data recorded for connected vehicles are used, and the driving behavior of 34,167 drivers is analyzed.

### 1.2. Method Used for Driving Style Classification

Additionally, three types of methods are proposed to recognize risky drivers. First, several clustering models have been established in different studies to cluster drivers into different driver types [10,15,34]. Different drivers are divided automatically based on the parameter differences [21]. Second, the category of drivers is marked by a questionnaire survey, and then a machine learning model, such as neural networks and support vector machines (SVMs), is established to classify drivers. Similar to the first method, the extraction of driving parameters or features is the key [23]. Third, parameter thresholds are set. For example, once the speed exceeds a certain value or the frequency of a sharp acceleration and deceleration exceeds a certain value, the drivers are considered aggressive drivers. However, this method is highly subjective [24,35]. The k-means method was used to classify driving styles in this study, which is a simple and efficient method.

### 1.3. Limitations in Previous Studies

Numerous studies have investigated how to recognize driving styles, and the characteristics of different types of drivers have been widely studied. Ref. [36] analyzed the driving styles of car-hailing drivers in different driving tasks (defined as cruising, ride requests, and drop-off). However, the in-depth analysis of the driving styles for temporal and spatial distributions needs to be supplemented. The reason is as follows: In previous research, there was an implicit assumption that the drivers’ driving style does not change over time when classifying. This means that the drivers’ driving style is their inherent attribute characteristic. However, studies have shown that the driving speed of drivers varies at different times of the week and day [37]. Because parameters such as the velocity are one of the important parameters describing driving style, the same driver in different time periods may have different styles. Furthermore, a driver responds differently to the different traffic scenarios [17]. For example, the research of Chengdu proves the impact of state-led suburbanization on traffic crash density [38]. At a micro level, compared with road segments, intersections are important areas of driving scenario changes when driving in a road network. Therefore, there will be some differences between the drivers’ driving behavior at intersections and in road segments. Driving styles through intersections will have distinctive characteristics [39].

Consequently, we use an economical but abundant data source (connected vehicle trajectory data) to classify the driving styles of online ride-hailing drivers to obtain the distribution of the driving styles of ride-hailing drivers in the city. The changes in driving styles at different periods are analyzed. Additionally, the driving characteristics of different types of drivers in intersections and road segments are analyzed quantitatively.

The contributions of this research are as follows:

(1) The driving styles of ride-hailing drivers are classified.
(2) The changes in the driving style of ride-hailing drivers are analyzed throughout the day.
(3) The driving characteristics of drivers with different driving styles are analyzed when passing road segments and intersections.

The remainder of the paper is organized as follows: Section 2 gives a description of the dataset and data preprocessing. Section 3 describes the research methods, including parameter choices, clustering methods, and the time division of the day. In Section 4, the results are analyzed. Section 5 concludes the paper.

## 2. Data

**Figure 1:**
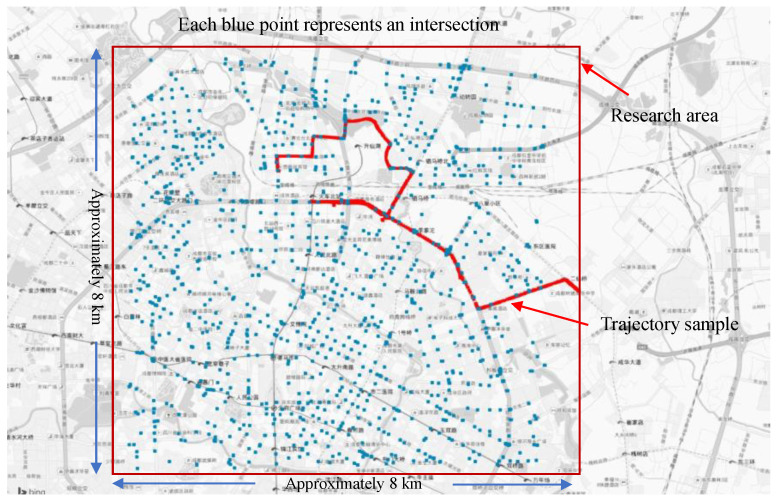
Trajectory data and intersection data.

The data were obtained from the Didi Chuxing Global Astrometric Interferometer for Astrophysics (GAIA) Open Dataset Initiative. This dataset is from the trajectory data of ride-hailing services within parts of Chengdu city on 1 November 2016. More than 31 million records, including 34,167 ride-hailing drivers, were recorded every 3 s. Sample data are shown in Figure 1. Chengdu is the capital of Sichuan Province and is a typical large city located in southwestern China. The city orientation, traffic characteristics, and geometric road design in Chengdu are similar to those in major Chinese cities. Our study area contains important ring roads, as well as radial roads, all of which are linear with orderly standards. Additionally, this dataset has been widely used in transportation research [40]. The recorded data include the driver ID so that we could analyze the driving characteristics of each driver. Therefore, these data are more suitable for this study. The fields of the data are described in Table 1.

Moreover, the location information of intersections in Chengdu can be obtained from Baidu Location-Based Services (http://lbsyun.baidu.com/, accessed on 26 March 2020). The location data of 1663 intersections were obtained within the research area, as shown in Figure 1. The fields contained in the data are described in Table 2.

The raw data contain the time and location information of each driver, so it is necessary to calculate the distance, interval time, speed, and acceleration of the two adjacent trajectory points for the parameter extraction of drivers. Since one value can be calculated from two records, we define the first value of these parameters to be 0 for each driver.

Although this dataset has a high accuracy, some outliers still exist. The noise cleaning processing is adopted. We removed some data that significantly exceeded the current vehicle’s acceleration or deceleration performance (less than −4 m/s^2^ or above 4 m/s^2^) and road speed limit (over 120 km/h ≈ 33.3 m/s). Since the first record of each driver is 0 (speed and acceleration), it was also removed. Finally, 98.07% of the data are valid. The 50–100 m radius around the center of the intersection was taken as the actual range of the intersection based on intersection levels. Therefore, we believe that the trajectory of connected vehicles within this range is generated by driving in intersections, and the remaining trajectory points are generated by driving in road segments. A global map-matching algorithm was applied to match the intersection information to the trajectory data [41]. Sample data are shown in Table 3.

According to the statistics of the data, trip records for ride-hailing drivers were available for 946 of the 1664 intersections. On average, each intersection has 2154 driver trajectory records. The average travel distance of each driver is 17.49 km, and the average travel time is 0.78 h.

**Figure 2:**
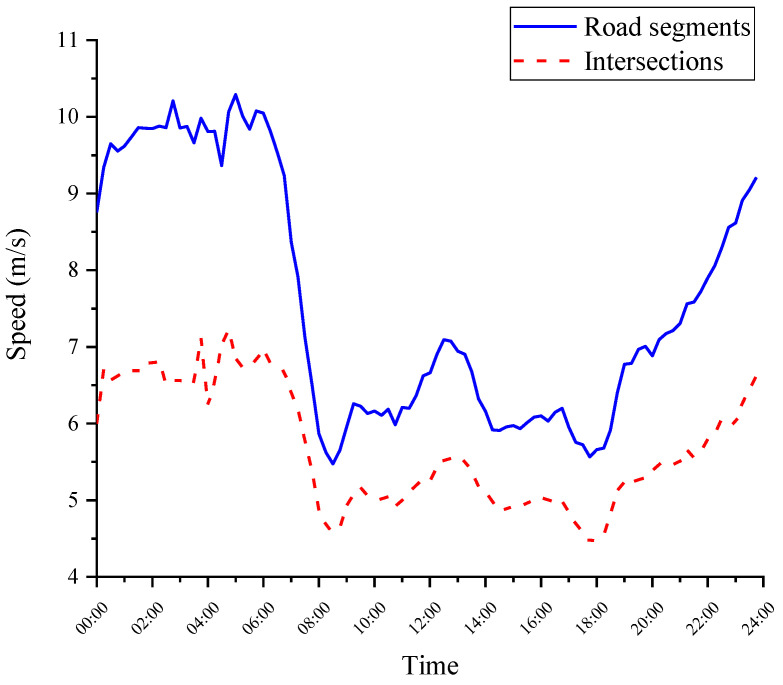
Average speed at intersections and in road segments.

An exploratory analysis of the data was performed, which included the speed distribution, acceleration, and deceleration distribution at different times at intersections and in road segments. From Figure 2, the speed at intersections is significantly lower than the road speed at any time. This result is mainly due to traffic lights, traffic conflicts, and pedestrian traffic at intersections, resulting in a reduced speed. Furthermore, the speed distribution clearly shows the peak characteristics in the morning and evening and at night (0–6 a.m.) when the speed is much higher than that during the day. This distribution is consistent with previous studies on the traffic characteristics of Chengdu [40].

**Figure 3:**
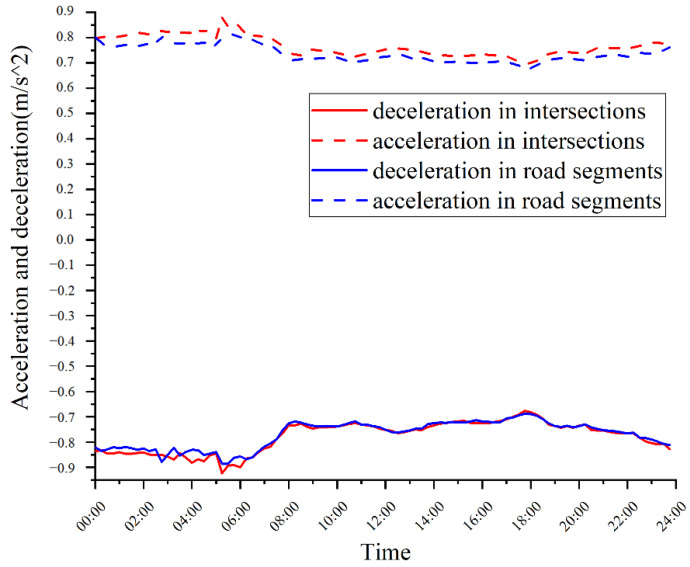
Average acceleration and deceleration at intersections and in road segments.

Additionally, the average acceleration and deceleration through intersections and road segments at different times were analyzed. As shown in Figure 3, the acceleration and deceleration values also change with time, with higher values at night. The acceleration at intersections is found to be greater than that in road segments at any time. Moreover, the data also reflect the asymmetric acceleration and deceleration characteristics, which means that the deceleration value is higher than the acceleration value, i.e., approximately 1.5% higher at intersections and 4.4% higher in road segments.

Based on the above analysis, we find that the speed, acceleration, and deceleration have time-varying and space-varying characteristics, and these parameters are the key parameters for describing driving behavior. Therefore, research on driving styles needs to consider temporal and spatial factors. Further analysis is described in Section 4.

## 3. Method

**Figure 4:**
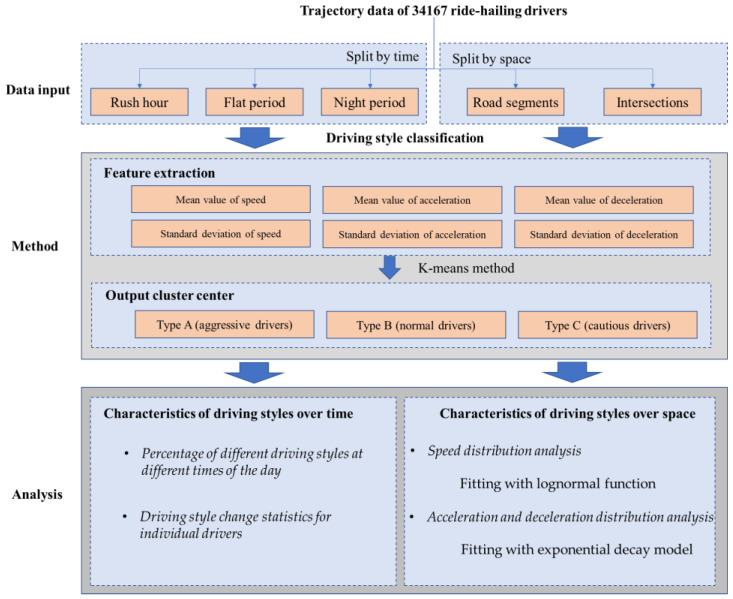
Flowchart of the research.

In Section 2, the characteristics of the overall data were analyzed. However, to study driving style, the characteristics of each driver are essential. First, driving features need to be extracted from the driving parameters, which were used to represent the driving style of a ride-hailing driver. Then, a method is needed to classify the driving styles of these drivers. We used an unsupervised learning clustering method, which is simple and efficient. A flow chart of this study is shown in Figure 4.

### 3.1. Measuring Driving Styles (Feature Extraction)

We selected three sets of parameters to describe the driving style of drivers: speed-related parameters, acceleration-related parameters, and deceleration-related parameters. The reasons are as follows.

First, velocity is one of the most frequently selected parameters as a driver feature from trajectory data [5,19,20,36,42,43]. In the same driving environment, if a driver tends to drive at a faster speed, then the driving style will be considered more aggressive [44]. However, even with the same average speed, the difference in the speed distribution also represents different driving styles. For example, two drivers A and B have the same average driving speed, but the speed of driver A is more variable than that of driver B, which means that A’s driving behavior is more stable and reliable, while B’s driving style is riskier. Therefore, the velocity and the standard deviation of velocity were chosen as features to represent the driving styles of drivers in this study [15].

Furthermore, risky drivers exhibit more dangerous driving events, such as more frequent and sharper acceleration [20]. The change in acceleration and deceleration is called “Jerky driving”, which is an important parameter reflecting the driver’s driving style [43,45]. If a driver tends to start or brake the vehicle with greater acceleration, then his or her driving characteristics are more unstable [18]. Therefore, the mean and standard deviation of the acceleration value were also taken as features in this study.

Similarly, we also used deceleration as a feature to describe the driving style of the driver. The reason for choosing the deceleration parameter is that the types of vehicles of ride-hailing drivers are not uniform, and different vehicles have different braking performances. Better braking performance means that the driver can react to events faster. Another important reason is, from a psychological point of view, to avoid collisions, the drivers decelerate more sharply than accelerating. This phenomenon is referred to as “driving behavior asymmetry” [46], which means that the deceleration and acceleration distributions are different [47,48]. Therefore, we take the acceleration value and its standard deviation as features.

Finally, six features are used to describe driver behavior and are expressed as $\mu_{s}$, $\sigma_{s}$, $\mu_{a}$, $\sigma_{a}$, $\mu_{d}$, and $\sigma_{d}$, where $\mu_{s}$, $\mu_{a}$, and $\mu_{d}$ denote the mean values of the speed, acceleration, and deceleration, respectively. Similarly, $\sigma_{s}$, $\sigma_{a}$, and $\sigma_{d}$ denote the standard deviation values of the speed, acceleration, and deceleration, respectively.

### 3.2. Clustering Method

Previous research [21] has summarized the methods of driving style classification, mainly supervised and unsupervised. Supervised classification methods include the SVM [20,21], Gaussian mixture model (GMM) [15], and various deep learning methods [49], which require labeled data. In this paper, we propose a concise identification that captures the difference between different drivers. An unsupervised classification method is more reasonable and can classify unknown data into categories.

Therefore, the k-means method was adopted to cluster the data, which has been widely used in previous studies [4,20]. This method possesses several advantages, such as algorithmic simplicity, a fast calculation speed, and a good clustering effect. Drivers are clustered into several types, in which each sampled record belongs to the cluster with the nearest mean. The cluster center values of the different categories reflect the distribution of the overall characteristic parameters. The key is to determine the value of *k*, the number of clusters. Generally, the elbow method is used to determine the optimal number of clusters. This method defines the squared distance error between the centers of the cluster and the sample points within the cluster as distortions, as shown in Equation (1), where $A_{c}$ denotes a cluster of c, *p* is the sample point in $A_{c}$, and $m_{c}$ is the center of $A_{c}$.

(1) $$SSE=\sum_{c=1}^{k}\sum_{p\in A_{c}}|p-m_{c}{|}^{2}$$

**Figure 5:**
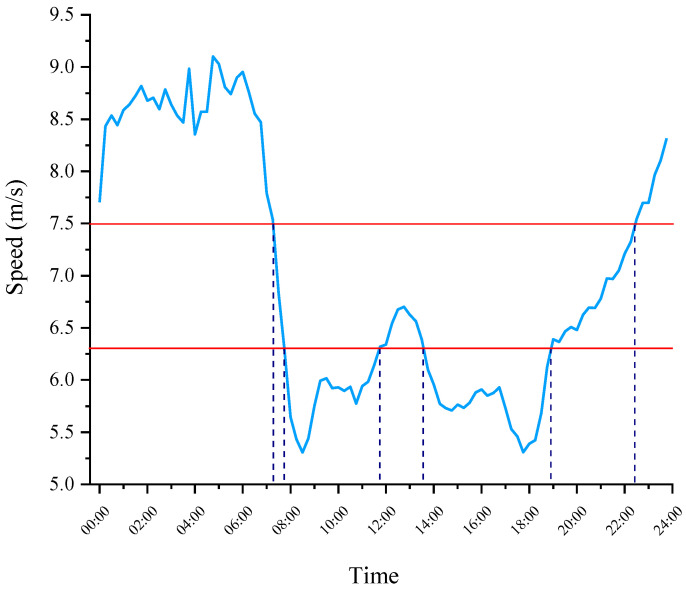
Time division based on speed.

The difference in the velocity distribution and acceleration distribution may be due to different traffic flow statuses instead of different driving styles. Therefore, the analysis should be carried out under the same traffic flow status. Generally, the data are divided into several time periods, assuming that the traffic state does not change within the same period. In a previous study, the whole day was uniformly divided into several periods or defined as a general peak and flat period [15,50]. There may be multiple traffic conditions in the same time period, such as morning and afternoon. For example, refs. [5,36] believed that 7:00 a.m.–10:00 a.m. is a peak period. However, as shown in the speed curve in Figure 2, this division of time does not fit this dataset. Different from previous studies, we divided the whole day into several discrete periods according to the distribution of the speed and divided the data with similar speed characteristics into the same group. The data were grouped by traffic flow status in this method. As shown in Figure 5, three groups of data were obtained. We defined the first group as rush hour, with speeds less than 6.3 m/s, and the periods were 7:45–11:30 and 13:45–18:45. The second group was defined as the flat period, with speeds ranging from 6.3 to 7.5 m/s. The time periods were 7:15–7:30, 11:45–13:30, and 19:00–22:15. Additionally, the third group was defined as the night period, with speeds above 7.5 m/s, and the periods were 22:30–24:00 and 00:00–7:30. Six features were extracted from the three groups of data and used for a cluster analysis. See Section 4 for the results.

## 4. Results and Discussion

### 4.1. Classification of Driving Styles of Ride-Hailing Drivers

**Figure 6:**
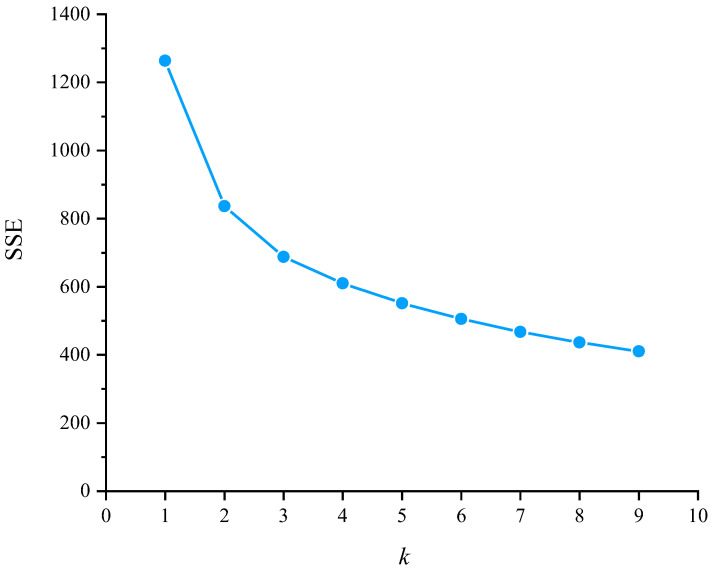
Variation in the SSE value with *k*.

The feature data of 34,167 drivers were applied to the k-means method, and the elbow rule was used to determine the optimal value of *k*. The results are shown in Figure 6. We can see from the results that when *k* ≥ 3, the sum of squares error (SSE) value changes more smoothly. Therefore, *k* = 3 was selected as the optimal clustering value.

As seen from Table 4, the data were clustered into three categories based on six features $\mu_{s}$, $\sigma_{s}$, $\mu_{a}$, $\sigma_{a}$, $\mu_{d}$, and $\sigma_{d}$. The clustering center values of all features in the three types show obvious characteristics from high to low. As described in previous studies [15,17], we defined three types of drivers, and the drivers of different types represented different risk levels.

**Type A:** The parameter values of this type are the highest, which means that this type of driver demonstrates the highest speed, a more discrete speed distribution, and a sharper acceleration and deceleration. Ref. [10] found that speeding was the most important driving behavior linked to risky behavior. Ref. [20] believed that risky drivers tend to have a larger acceleration. This type of driver shows higher risk characteristics. Therefore, we define drivers in this group as **aggressive drivers**.

**Type B:** Compared to Type A drivers, drivers in this group have more average speed and acceleration, and the distribution is more concentrated [51]. We define drivers in this group as **normal drivers**.

**Type C:** Drivers in this group have the lowest parameter value, which means drivers drive slowly and the acceleration is smooth. Drivers in this group are defined as **cautious** **drivers**.

The clustering result of driving style was obtained by synthesizing the driving parameters of drivers throughout the day, which represents a ride-hailing driver’s driving risk level in a day. It can be seen that the six parameter values of Type A are the largest, the parameter values of Type B are in the middle, and the parameter values of Type C are the smallest. This result is similar to the results of [17,36] and proves the reliability of our results. The proportions of aggressive drivers, normal drivers, and cautious drivers were 19%, 54%, and 27%, respectively. Compared with the study by [36], where more than 24.9% of professional drivers are aggressive, our result shows that only 19% of drivers are aggressive. The possible reason is that Chengdu has implemented a strict management policy on online ride-hailing. This policy requires that all ride-hailing vehicles install satellite positioning devices with a driving record function and data access to the government supervision platform (http://gk.chengdu.gov.cn/govInfoPub/detail.action?id=85649&tn=6, accessed on 18 November 2016). In this way, drivers will pay more attention to the passenger’s ride experience and the star rating after the ride. The results regarding the proportions of different types of drivers indicate that ride-hailing drivers may provide a more conservative driving style to provide passengers with a more comfortable ride experience under strict management policies [5].

### 4.2. Characteristics of Driving Styles over Time

Based on the division of time in Section 3, the descriptive statistics of six parameters in different time periods were calculated, as shown in Table 5. From the data in different time periods, in addition to the different speed mean values, the standard deviation distribution is also different and larger, especially at night. This change shows that there are great differences in individual driving behavior at night. Possible reasons for this result are that traffic flows more smoothly at night, so driving speeds are faster, but drivers tend to accelerate and decelerate more sharply to avoid collisions due to the limitations of their driving vision [52,53]. During the peak period, the driver’s driving parameters exhibit the least difference due to the restrictions of road traffic conditions. The data show changes in driving parameters throughout the day, which means that it is more reasonable to evaluate the driving style of drivers in different periods. In this section, the change in a driver’s driving style is tracked at different times. For example, if a driver shows a more aggressive driving style than other drivers, whether in peak or flat periods, then the platform needs to pay special attention to the ride-hailing driver.

We also applied the k-means method to cluster drivers in different periods. This method was used to analyze each driver’s driving style changes with the change in the traffic state. Similarly, we set *k* = 3, and the clustering results are shown in Table 6.

From Table 6, the driving parameters of the same type of driver are different in different time periods. This change occurs because we divided the data according to the traffic state to ensure that the analysis of driving style represents the same traffic status. For an aggressive driver, even if he or she is aggressive, his or her driving parameters will not change more than at night because the driving freedom at night is greater. In different periods of time, the drivers of the three driving styles all show similar characteristics; that is, each parameter of the Type A driver demonstrates the largest value (the maximum speed, the most frequent acceleration and deceleration, the largest acceleration and deceleration, etc.).

We also found that the distribution of different types of drivers was different in the three time periods. The proportion of Type A ride-hailing drivers at night was significantly higher than that at other periods. One possible reason is that drivers who provide ride-hailing services at night are more professional ride-hailing drivers, and they are more inclined to complete the service quickly to increase their income [5]. On the one hand, the traffic volume is relatively small at night, and driving fast at night occurs more frequently than during the day. The different proportions of aggressive drivers in different periods also imply that the risk level of the road network traffic is different. This result also explains to a certain extent why the risk degree is higher at night from the perspective of driving style [53].

**Figure 7:**
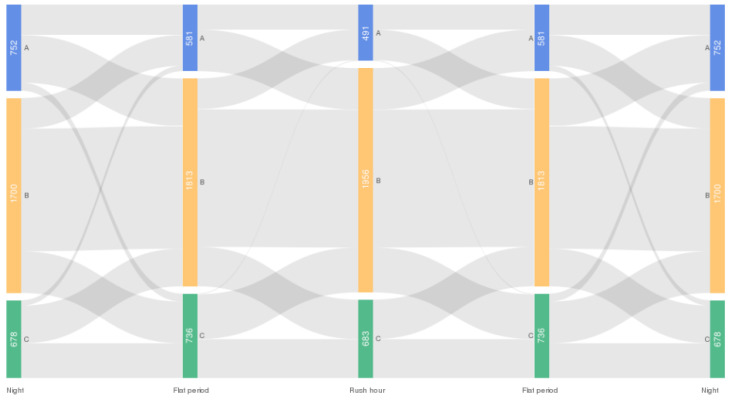
Driving style shifts of ride-hailing drivers.

The proportion of driving styles changes over time, which indicates that there are some drivers who belong to Type A during rush hour and Type B at night. We understand how many drivers will change their driving styles over time, which is helpful for assessing how many mature drivers are in the road network and providing targeted training for ride-hailing drivers. Because skilled drivers generally maintain a stable driving style, a more stable driving style helps improve traffic efficiency and safety and reduce energy consumption [6,54]. The change in driving style of drivers over time is shown with a Sankey diagram (Figure 7). Notably, although there were 34,167 drivers for the whole day, only 3130 drivers were recorded in all three periods. Therefore, data recorded from drivers during all periods are necessary to analyze driving style shifts over time.

From Figure 7, we can see that 31.79% of the drivers did not change their driving style in different periods. Among them, 13.3% of drivers maintained a high risk, and 18.3% of drivers maintained a low risk. These two types of drivers can be considered skilled ride-hailing drivers because they maintain a relatively stable driving state. For aggressive drivers who maintain stability, we recommend that the platform provide new driving training or reduce the number of orders to ensure the safety of service. There were also a small number of drivers (3.93%) whose driving style changed significantly, such as from Type A to Type C or from Type C to Type A. This type of driver can be defined as an unstable type, which should be a focus. For example, the platform can remind these drivers that their driving style has changed too much during specific time periods and to not drive aggressively, or the platform can reduce the number of orders during these time periods. More individual information data should be used to analyze the reasons for this change. In this study, we did not explore the causes. Most drivers’ driving styles fluctuate in different periods, such as switching between Type A and Type B or Type B and Type C. This change may be caused by the impact of the traffic status [17]. Drivers have different sensitivities to changes in traffic conditions, resulting in subtle fluctuations in driving style.

The results of this section show that only some of the ride-hailing drivers in the road network can guarantee the same driving style in different traffic conditions. For example, some drivers are aggressive or conservative from morning to night. It is very likely that these drivers have richer driving experiences and skills because skilled drivers will maintain a stable driving style [55,56]. The volatility of the driving style from rush hour to the flat period is the greatest. This effect is because most drivers have a greater probability of changing their driving style when the traffic environment changes. From rush hour to the flat period, the number of vehicles in the road network has drastically decreased, causing drivers to be more conservative or more aggressive.

### 4.3. Driving Characteristic Analysis of Drivers with Different Driving Styles on Road Segments and Intersections

Section 4.2 revealed that the driving style will change over time, which is mainly due to different responses of drivers in different traffic environments. To further analyze the changes in the driving parameters of drivers as a function of driving style in different traffic environments, we divide the road network into road segments and intersections. This division is performed because the largest change in the driving environment in the road network is the switch between roads and intersections. In road segments, car following and lane change behavior are the most important driving operations that need to be considered. However, more uncertain factors should be considered, such as pedestrian crossings and signal lights at intersections. Therefore, the driving parameter distribution of different types of drivers at intersections and in road segments needs to be explored, including the speed distribution and acceleration distribution in this section.

Notably, in this section, we discuss the driving parameter changes in different driving styles, where the driving style we adopt is the clustering result throughout the day, i.e., the result of Section 4.1. The reason is that although the driving parameters are different in different time periods, the parameter variation is consistent in the same time period, which means we can observe the same characteristics at different time periods.

#### 4.3.1. Speed Distribution

**Figure 8:**
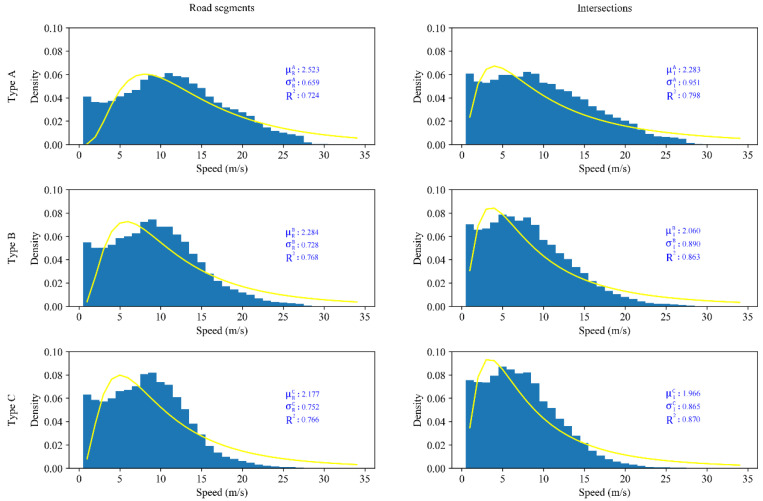
Speed distributions for different types of drivers in different driving environments.

First, the speed distribution was analyzed. Speed distribution histograms of different types of drivers at road segments and intersections were plotted, and a lognormal function Equation (2) was used to fit the speed distribution, where *x* denotes the speed, *μ* is the scale parameter (the mean of the log of the distribution), and *σ* is the shape parameter (and is the standard deviation of the log of the distribution). The fitting curve is shown in Figure 8.

(2) $$p(x)=\frac{1}{x\sigma \sqrt{2\pi }}e^{-\frac{{\left(\ln x-\mu \right)}^{2}}{2\sigma^{2}}}$$

A high value of *R*^2^ (ranging from 0 to 1) represents a good fitting model. The results show that the *R*^2^ values of the six fittings are all greater than 0.7, which indicates that the lognormal model could represent more than 70% of the features of the distribution. The Kolmogorov–Smirnov (K-S) test was employed to test whether data come from the same distribution. The results show that the *p* value is lower than 0.05, which proves that each group of data comes from a completely different distribution. This result means that the fitting parameters of different groups can be compared with each other. To facilitate the subsequent analysis, we define $\mu_{R}^{A}$ and $\mu_{I}^{A}$ as the mean values and $\sigma_{R}^{A}$ and $\sigma_{I}^{A}$ as the standard deviations of the speed distributions of Type A drivers on road segments and intersections, respectively. The corresponding $\mu_{R}^{B}$, $\mu_{I}^{B}$, $\sigma_{R}^{B}$, and $\sigma_{I}^{B}$ are defined as the fitting parameters of the speed distributions of Type B drivers, and $\mu_{R}^{C}$, $\mu_{I}^{C}$, $\sigma_{R}^{C}$, and $\sigma_{I}^{C}$ are speed fitting parameters of Type C drivers. The fitting parameters are shown in Table 7. The detailed analysis is as follows.

According to the characteristics of the lognormal distribution, the effect of the scale parameter is to stretch or squeeze the graph. The effect of the location parameter is to translate the graph. If the value of *μ* is larger and the curve is more to the right, then the speed is more distributed with a larger value. The larger the value of σ is, the more discrete the speed distribution [57].

Table 7 shows that drivers of the same type of driving style tend to have higher speeds in the road sections than at the intersections; that is, the μ value is larger ($\mu_{R}^{A}$ *>* $\mu_{I}^{A}$*,* $\mu_{R}^{B}$ *>* $\mu_{I}^{B}$*,* $\mu_{R}^{C}$ *>* $\mu_{I}^{C}$). The value of σ has opposite characteristics ($\sigma_{R}^{A}$ *<* $\sigma_{I}^{A}$*,* $\sigma_{R}^{B}$ *<* $\sigma_{I}^{B}$*,* $\sigma_{R}^{C}$ *<* $\sigma_{I}^{C}$). The main reason for this phenomenon is the complex driving environment of intersections, such as traffic light shifts and deceleration to avoid pedestrians, which cause frequent changes in the speed or driving at a low speed. We also found that drivers with a higher degree of risk are more inclined to keep driving at high speeds, whether they are driving on roads or intersections ($\mu_{R}^{A}$ *>* $\mu_{R}^{B}$ *>* $\mu_{R}^{C}$*,* $\mu_{I}^{A}$ *>* $\mu_{I}^{B}$ *>* $\mu_{I}^{C}$).

Another interesting finding is revealed. For the parameter σ, the higher the risk of the driver is, the smaller the value of σ in road segments, while in the intersections, the opposite characteristics are observed ($\sigma_{R}^{A}$ *<* $\sigma_{R}^{B}$ *<* $\sigma_{R}^{C}$*,* $\sigma_{I}^{A}$ *>* $\sigma_{I}^{B}$ *>* $\sigma_{I}^{C}$). This finding means that high-risk drivers will maintain a higher and more stable speed in the road sections than at the intersections. On the other hand, conservative drivers tend to adopt more conservative strategies in complex traffic environments, such as steady low-speed driving [58]. According to the research of [55,56], drivers with rich driving experience will have a higher degree of risk, such as faster speeds. Therefore, this finding may be explained by the fact that most high-risk drivers are experienced drivers. From the speed distribution, we further found that high-risk drivers not only exhibit faster speeds but also have different characteristics of the dispersion degree of their speed distribution in road segments and intersections. The analysis result also implies that aggressive drivers show more aggressive driving styles in road segments, and conservative drivers have more conservative driving styles at intersections.

#### 4.3.2. Acceleration and Deceleration Distribution

**Figure 9:**
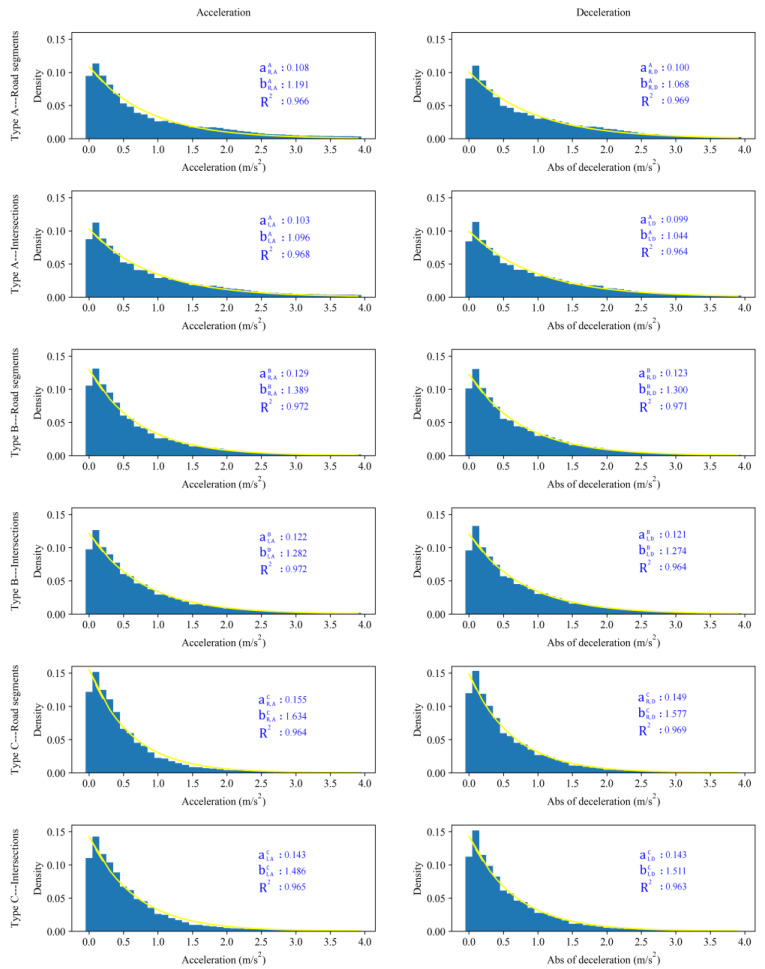
Acceleration and deceleration for different types of drivers in different driving environments.

Then, the distributions of acceleration and deceleration in road segments and the intersection of different driving styles were analyzed, and an exponential decay model Equation (3) was applied to fit the distributions, where *a* denotes the initial quantity and *b* denotes the decay constant. The fitting results show that *R*^2^ is greater than 0.95 in all cases, and 12 distributions are significantly different, as the *p* value of the K-S test is lower than 0.05. The results are shown in Table 8. The fitted curve is shown in Figure 9.

(3) $$y=ae^{-bx}$$

The most important parameter affecting the exponential model is the value of *b* (the scale parameter). The larger parameter *b* is, the closer the curve is to the coordinate axis, which means that the acceleration and deceleration values are concentrated at smaller values. To facilitate the subsequent analysis, we define $a_{R,A}^{A}$, $b_{R,A}^{A}$, $a_{R,D}^{A}$, and $b_{R,D}^{A}$ as the acceleration and deceleration distribution fitting parameters of the Type A driver in the road segments. Correspondingly, $a_{I,A}^{A}$, $b_{I,A}^{A}$, $a_{I,D}^{A}$, and $b_{I,D}^{A}$ are defined as the acceleration and deceleration distribution fitting parameters of the Type A driver at the intersections. $a_{R,A}^{B}$, $b_{R,A}^{B}$, $a_{R,D}^{B}$, and $b_{R,D}^{B}$, $a_{I,A}^{B}$, $b_{I,A}^{B}$, $a_{I,D}^{B}$, and $b_{I,D}^{B}$ are the fitting parameters of Type B drivers in the road segments and at the intersections. $a_{R,A}^{C}$, $b_{R,A}^{C}$, $a_{R,D}^{C}$, and $b_{R,D}^{C}$, $a_{I,A}^{C}$, $b_{I,A}^{C}$, $a_{I,D}^{C}$, and $b_{I,D}^{C}$ are the fitting parameters of Type C drivers.

From Table 8, first, we can see that for the same type of drivers, their acceleration or deceleration values are distributed differently in road segments and at intersections: $b_{I,A}^{A}$ < $b_{R,A}^{A}$, $b_{I,D}^{A}$ < $b_{R,D}^{A}$, $b_{I,A}^{B}$, < $b_{R,A}^{B}$, $b_{I,D}^{B}$ < $b_{R,D}^{B}$, $b_{I,A}^{C}$ < $b_{R,A}^{C}$, and $b_{I,D}^{C}$ < $b_{R,D}^{C}$. For example, Type A drivers tend to demonstrate greater acceleration or deceleration at intersections. This behavior is caused by the complex environment of the intersection. In contrast to road segments, the smoothness of driving at intersections cannot be guaranteed. This result also indicates that intersections are riskier than road segments.

Second, we also found that the deceleration distribution of the same type of drivers is greater than the acceleration distribution whether they are driving on road segments or intersections ($b_{R,A}^{A}>b_{R,D}^{A}$, $b_{I,A}^{A}>b_{I,D}^{A}$, $b_{R,A}^{B}>b_{R,D}^{B}$, $b_{I,A}^{B}>b_{I,D}^{B}$, $b_{R,A}^{C}>b_{R,D}^{C}$). This phenomenon is called asymmetric driving behavior [46,47,48]. This driving behavior has been found in previous studies and is an important method for explaining the evolution characteristics of macroscopic and microscopic traffic flows [58]. In particular, we found that there is a special situation where $b_{I,A}^{C}<b_{I,D}^{C}$, that is, a conservative driver whose acceleration value is greater than deceleration at an intersection. This phenomenon can be explained by the fact that conservative drivers prefer to maintain a lower speed and seldom perform a sharp deceleration to avoid staying in a complex driving environment. Once the driving conditions permit, they will accelerate through the intersection. We inferred that this result is most likely because conservative drivers are mostly drivers with less driving experience. Previous studies have found that driving experience significantly affects driving safety, with experienced drivers being at greater risk [55,56,59]. More driver information data are needed to prove this inference. These findings imply that different distributions of different types of drivers on the road network will also profoundly affect the characteristics of traffic flow. This phenomenon should be taken into account in future research on traffic flow.

These findings all reveal that drivers driving at intersections exhibit driving behaviors that are different from behaviors exhibited when driving in road segments. Different types of drivers also reflect different driving characteristics in different driving environments. Previous studies have found that cautious behavior can reduce the occurrence of accidents [60]. Our study explains the meaning of “Cautious behavior” to some extent, which means that we more deeply reveal the reasons for the frequent occurrence of accidents at intersections from the perspective of driving behavior. Therefore, the results have a positive effect on further explaining why traffic accidents occur frequently at intersections.

## 5. Conclusions

In this study, the driving styles of drivers were analyzed based on substantial trajectory data. Taking the mean and standard deviation of the velocity, acceleration, and deceleration as the features, k-means clustering was adopted to cluster the drivers. The results show that these six features can cluster drivers into three types at different risk levels.

We tracked the driver’s driving style in different time periods. Only 31.79% of drivers had a stable driving style, which did not change with time. There were also a small number of drivers (3.93%) whose driving style changed significantly, and most drivers’ behaviors were relatively stable, which means there was a slight change in the risk level. In contrast to previous studies, we quantitatively discovered the proportion of drivers with different driving styles at different times in the road network, as well as the ratio of switching between styles, while explaining the reasons for switching between different styles. At the macro level, the proposed method provides new ideas for further assessing the degree of risk to the road network, and at the micro level, it provides a reference value for analyzing the driving performance of ride-hailing drivers.

The driving parameters of different types of drivers in road segments and at intersections were analyzed, including the speed, acceleration, and deceleration distributions. The lognormal model and exponential decay model were applied. The results show that conservative drivers cross intersections with more conservative driving styles (maintain a steady low speed), and aggressive drivers have more aggressive driving styles on road segments (maintain a steady high speed). From the perspective of the acceleration and deceleration distributions, we find that the driver’s deceleration is generally greater than the acceleration. The existence of asymmetric driving behavior is proven from the trajectory data. However, asymmetric driving behaviors present different characteristics in road segments and at intersections for conservative drivers. All types of drivers exhibit greater acceleration and deceleration at intersections than in road segments. High-risk drivers have a higher probability of sharp braking and acceleration. This result means that intersections are more dangerous in space. High-risk drivers should obtain the necessary reminders.

The findings from this study have several important implications. First, the driving style of ride-hailing drivers should be a time-based assessment. The proportion of risky drivers in different periods leads to different risk coefficients on the road, which could explain the difference in the distribution of accident proportions over time. Second, the risk coefficient of intersections is higher than that of road segments, which also provides a spatial analysis of accidents. Finally, for different types of ride-hailing drivers, different driving suggestions should be provided by ride-hailing platforms for specific times and spaces and should be utilized to design training policies for ride-hailing drivers.

This study could be improved upon and further evaluated with regard to the following points. In this research, only one day of data was used for the analysis, while long-term observations may be more accurate, such as using data from one week, one month, or even years. Additionally, considering geographical factors and nearby floating population in the method may give us more new findings. In the future, the reasons for driver style changes over time and space will be analyzed from the perspectives of physiology and psychology through experiments and questionnaire surveys, and more personal characteristics of drivers will be used.

## Figures and Tables

**Table 1:** Trajectory data.

Field	Type	Sample a	Comment
Driver ID	String	Glox.jrrlltBMvCh8nx	Anonymized
Order ID	String	Jkkt8kxniovFuns9qrrl	Anonymized
Timestamp	String	1,501,584,540	Unix Timestamp, in seconds
Longitude	String	104.04392	GCJ-02 Coordinate System
Latitude	String	30.705191	GCJ-02 Coordinate System

All trajectory points are only in the range from [30.727818, 104.043333] and [30.726490, 104.129076] to [30.655191, 104.129591] and [30.652828, 104.042102].

**Table 2:** Intersection location data.

Field	Type	Sample a	Comment
Intersection ID	String	691	Increment
Center Latitude	String	30.68583438	GCJ-02 Coordinate System
Center Longitude	String	104.101755	GCJ-02 Coordinate System
Name	String	Ren Ju Intersection	-

**Table 3:** Sample data.

Driver_id	Timestamp	Longitude	Latitude	Interval Time (s)	Velocity (m/s)	Acceleration (m/s^2^)	Intersection_id
14c668b0819db37d6c38fbddd33f33da	9:52:12	104.0872	30.65411	0	0.00	0.00	N
14c668b0819db37d6c38fbddd33f33da	9:52:15	104.0869	30.65423	3	9.69	0.00	N
14c668b0819db37d6c38fbddd33f33da	9:52:21	104.0863	30.65452	6	11.25	0.26	N
14c668b0819db37d6c38fbddd33f33da	9:52:24	104.086	30.65468	3	12.64	0.46	440
14c668b0819db37d6c38fbddd33f33da	9:52:27	104.0856	30.65487	3	14.01	0.46	440
14c668b0819db37d6c38fbddd33f33da	9:52:30	104.0852	30.65503	3	12.36	−0.55	N
14c668b0819db37d6c38fbddd33f33da	9:52:33	104.0849	30.65518	3	11.34	−0.34	N
14c668b0819db37d6c38fbddd33f33da	9:52:36	104.0847	30.6553	3	9.13	−0.74	N

**Table 4:** Clustering results of driving styles (cluster center).

	Cluster	Type A (N = 6522)	Type B (N = 18,427)	Type C (N = 9218)
Feature				
$\mu_{s}$ (m/s)		11.32	8.46	7.46
$\sigma_{s}$ (m/s)		6.17	5.15	4.24
$\mu_{a}$ (m/s^2^)		0.96	0.75	0.59
$\sigma_{a}$ (m/s^2^)		0.88	0.74	0.56
$\mu_{d}$ (m/s^2^)		0.98	0.76	0.61
$\sigma_{d}$ (m/s^2^)		0.86	0.71	0.56

**Table 5:** Descriptive statistics of driving features at different time periods.

Features	Rush Hour N = 24,379				Flat Period N = 20,967				Night Period N = 9979			
	Min	Max	Mean	SD	Min	Max	Mean	SD	Min	Max	Mean	SD
$\mu_{s}$ (m/s)	0.384	24.494	8.064	2.144	0.637	23.767	8.911	2.423	0.371	25.273	10.492	2.630
$\sigma_{s}$ (m/s)	0.000	13.048	4.951	1.040	0.000	12.797	4.908	1.097	0.000	11.332	5.135	1.130
$\mu_{a}$ (m/s^2^)	0.000	3.899	0.732	0.199	0.003	3.772	0.756	0.224	0.013	3.325	0.795	0.243
$\sigma_{a}$ (m/s^2^)	0.000	1.507	0.702	0.149	0.000	1.726	0.712	0.170	0.000	1.683	0.734	0.183
$\mu_{d}$ (m/s^2^)	0.000	3.966	0.735	0.186	0.032	3.718	0.769	0.211	0.000	3.961	0.833	0.241
$\sigma_{d}$ (m/s^2^)	0.000	1.677	0.679	0.137	0.000	1.701	0.697	0.152	0.000	1.786	0.740	0.169

**Table 6:** Clustering results of driving styles for different time periods (cluster center).

Features	Rush Hour			Flat Period			Night Period		
	N = 24,379			N = 20,967			N = 9979		
	Type A	Type B	Type C	Type A	Type B	Type C	Type A	Type B	Type C
$\mu_{s}$ (m/s)	10.79	7.81	6.92	12.18	8.46	7.7	12.75	9.73	9.35
$\sigma_{s}$ (m/s)	6.17	4.99	4.11	6.14	4.94	4.06	6.08	5.03	4.13
$\mu_{a}$ (m/s^2^)	0.95	0.74	0.58	0.96	0.78	0.58	0.98	0.79	0.56
$\sigma_{a}$ (m/s^2^)	0.87	0.73	0.54	0.87	0.75	0.53	0.89	0.75	0.49
$\mu_{d}$ (m/s^2^)	0.95	0.74	0.59	0.99	0.78	0.6	1.02	0.82	0.61
$\sigma_{d}$ (m/s^2^)	0.85	0.70	0.54	0.86	0.72	0.54	0.88	0.75	0.54
Drivers	4018	13,892	6469	3741	11,424	5802	2761	5141	2077
Rate	16%	57%	27%	18%	54%	28%	28%	51%	21%

**Table 7:** The speed distribution fitting parameters of different types of drivers.

Driving Style	Road Segments			Intersections		
	*μ* (m/s)	*σ* (m/s^2^)	*R* ^2^	*μ* (m/s)	*σ* (m/s^2^)	*R* ^2^
**Type A**	2.523	0.659	0.724	2.283	0.951	0.798
**Type B**	2.284	0.728	0.768	2.060	0.890	0.863
**Type C**	2.177	0.752	0.766	1.966	0.865	0.870

**Table 8:** The acceleration and deceleration distribution fitting parameters.

	Acceleration						Deceleration					
	Road Segments			Intersections			Road Segments			Intersections		
	*a*	*b*	*R* ^2^	*a*	*b*	*R* ^2^	*a*	*b*	*R* ^2^	*a*	*b*	*R* ^2^
**Type A**	0.108	1.191	0.966	0.103	1.096	0.968	0.100	1.068	0.969	0.099	1.044	0.964
**Type B**	0.129	1.389	0.972	0.122	1.282	0.972	0.123	1.300	0.971	0.121	1.274	0.964
**Type C**	0.155	1.634	0.964	0.143	1.486	0.965	0.149	1.577	0.967	0.143	1.511	0.963

## Data Availability

The data presented in this study are available on request from the corresponding author.
